# Does Individual Gambling Behavior Vary across Gambling Venues with Differing Numbers of Terminals? An Empirical Real-World Study using Player Account Data

**DOI:** 10.3389/fpsyg.2018.00158

**Published:** 2018-02-16

**Authors:** Dominic Sagoe, Ståle Pallesen, Mark D. Griffiths, Rune A. Mentzoni, Tony Leino

**Affiliations:** ^1^Department of Psychosocial Science, University of Bergen, Bergen, Norway; ^2^International Gaming Research Unit, Department of Psychology, Nottingham Trent University, Nottingham, United Kingdom; ^3^Department of Clinical Psychology, University of Bergen, Bergen, Norway

**Keywords:** casino environment, electronic gaming machines, gambling environment, gambling terminals, gambling venue, social facilitation

## Abstract

Research examining gambling behavior via experiments, self-report, and/or observation presents many methodical challenges particularly in relation to objectivity. However, the use of player account-based gambling data provides purely objective data. Based on this real-world data, the primary aim of the present study was to examine gambling behavior in gambling venues with different numbers of gambling terminals (i.e., venues with one terminal; 2–5 terminals; 6–10 terminals; 11–16 terminals). Player account-based gambling data aggregated over a year (2015) amounting to 153,379 observations within 93,034 individual gamblers (males = 74%; mean age = 44.1, *SD* = 16.4 years) were analyzed. Gambling frequency was highest in venues with 2–5 terminals (54.5%) and lowest in venues with 11–16 terminals (1.6%). Approximately half of the sample (52.5%) gambled in only one venue category, with the majority (81.5%) preferring venues with 2–5 terminals present. Only 0.8% of the sample gambled in all four venue categories. Compared to venues with one terminal, venues with two or more terminals were associated with gamblers placing more bets, and spending more time and money per session. However, gamblers had higher losses (albeit small) in venues with one terminal compared to venues with 2–5 terminals. No differences in net outcome were found between venues with one terminal and those with 6–10 and 11–16 terminals. Overall, the present study demonstrates that in the natural gambling environment, gambling behavior is reinforced in venues with multiple terminals.

## Introduction

### The gambling environment and gambling behavior

The “availability hypothesis” suggests that the greater the number of opportunities to gamble, the greater the amount of gambling (Orford, 2002). However, this hypothesis tends to be applied at a macro (i.e., societal) level rather than a micro (e.g., gambling venue) level. Additionally, while gambling in social venues such as casinos and pubs (usually with multiple terminals), wins are often announced to others in the venue through accompanying stimuli or cues such as lights and sounds coming from slot machines, as well as gamblers' reactions to wins and losses (Griffiths and Parke, 2003).

Consequently, the influence of various aspects of the gambling environment on gambling behavior has attracted the attention of researchers. On a simulated electronic gaming machine (EGM) task, individuals who received audio-visual feedback (such as winning bells and messages) from other gamblers (who were actually not present) placed more bets and lost more money in comparison to individuals who did not receive such audio-visual feedback (Rockloff and Dyer, 2007). In another laboratory-based study, roulette gamblers placed more chips and engaged in riskier betting behaviors when gambling with others compared to those who gambled alone (Cole et al., 2011). There is also experimental evidence that gambling with others increases betting speed (Brevers et al., 2015) and is associated with higher losses (Molde et al., 2017).

In contrast, on a simulated experimental EGM task, individuals gambling alone placed higher bets compared to those gambling in front of a perceived audience of 26 onlookers (Rockloff and Greer, 2011). The researchers also found that individuals gambling alone had less money left after finishing their gambling session compared to individuals gambling with a perceived audience of six onlookers. In another experimental study, individuals gambling alone played more trials and placed faster bets compared to individuals gambling with familiar or unfamiliar co-gamblers (Molde et al., 2017). Based on the aforementioned studies, empirical evidence relating to the influence of environmental factors on gambling behavior is mixed.

### The number of gambling terminals and gambling behavior

One aspect of the gambling environment literature concerns differences in gambling behavior across venues with differing numbers of gambling terminals. However, there are few studies on the topic and most of the available studies have been based on either self-report surveys or experimental laboratory studies. In one study, Haw and Hing (2011) conducted a telephone survey of 175 gamblers. Gamblers' favorite venues were categorized into a casino, club, or hotel according to higher venue size and number of available EGMs (although the authors did not present the specific numbers of EGMs available per venue). It was found that although casino patrons gambled less frequently, they had a higher monetary expenditure per visit than those whose favorite venues were relatively small clubs or hotels.

In a postal survey of 7,041 gamblers (Young et al., 2012), favorite venues were categorized into: casinos (median EGMs = 417 [289–544]), clubs (median EGMs: supermarket-attached = 45 [13–45]; peripheral = 16 [3–45]), and pubs (peripheral, and agglomerated: EGMs: *n* = 10). Results indicated that average EGM gambling time per session is highest for casinos (130.3 min), followed by clubs (supermarket: 63.9 min; peripheral: 62.4 min), and pubs (peripheral: 54.3 min; agglomerated: 37.7 min). In addition, casino and supermarket-attached gamblers had the highest associations with problem gambling.

Another study by Sévigny et al. (2016) compared 209 gamblers whose favorite venues were a gambling hall (*n* = 66; EGMs = 335) to others who mostly gambled in small venues (*n* = 143; EGMs = 5 or 10). Findings indicated that small venue patrons gambled more sessions per month but played fewer hours per session (although no per month difference was detected). Furthermore, small venue gamblers were about four times more likely to be associated with problem gambling consistent with previous findings (Clarke et al., 2012). It was also found that small venue gamblers had participated in gambling for more years than gambling hall patrons in line with previous evidence (Franco et al., 2011). Small venue gamblers also spent more money per hour (although neither per session nor per month differences were detected).

While the aforementioned studies provide insight into differences in gambling behavior across venues with different numbers of terminals, they are associated with methodical differences that may account for the mixed findings, as shown in a previous study that combined a focus group and an experimental laboratory study (Ladouceur et al., 2005). Additionally, the validity of self-reported data can be problematic especially when not objectively validated. Experimental laboratory-based studies of gambling behavior have also been criticized for their ecological limitations such as the absence of personal monetary risk or loss associated with real-world gambling (Lyons, 2006; Gainsbury and Blaszczynski, 2011). Thus, in order to draw stronger conclusions that would be more ecologically valid and generalizable, it is crucial that research on this topic is extended to more valid data collected from natural gambling environments.

### Player account-based gambling data

One innovative way through which gambling behavior can now be observed and studied is the use of player account-based gambling (behavioral tracking) data (Griffiths and Whitty, 2010; Gainsbury, 2011; Griffiths, 2014). Player account-based gambling data (whether via online gambling accounts or via loyalty card/player card data) can provide a novel and rich resource for investigations of gambling behavior in natural gambling environments. A major strength of player account-based gambling data is the unobtrusive and objective manner in which it is collected thereby overcoming observer- and subject-expectancy effects associated with experimental or laboratory studies (Griffiths, 2014). Another strength is their strong ecological validity as they are collected in the natural gambling environment.

Player account-based gambling data has been used in some recent gambling studies investigating, for instance, the association between structural game characteristics and gambling behavior (Leino et al., 2015), the effects of negative wins on gambling behavior (Leino et al., 2016), and gambling behavior among internet-based poker problem gamblers (Luquiens et al., 2016). It has also been used to examine the effectiveness of responsible gambling features among online gamblers including limit setting (Auer and Griffiths, 2013), pop-up messaging (Auer et al., 2014; Auer and Griffiths, 2015a), and personalized feedback (Auer and Griffiths, 2015b, 2016).

### The present study

Due to the methodical limitations of previous studies, the present study used player account-based gambling data. More specifically, the present (arguably pioneering) study investigated individuals' gambling behavior across gambling venues with differing numbers of terminals (using the availability of multiple terminals as an environmental factor or proxy for congregations of gamblers). As noted previously, the few studies on gambling behavior across venues with differing numbers of gambling terminals have produced mixed findings making hypothetical inferences difficult to draw. Nonetheless, in line with previous findings (e.g., Young et al., 2012), it was expected that individual gambling behavior would vary as a function of the number of terminals in a venue. More specifically, it was expected that compared to venues with one terminal, venues with two or more terminals would be associated with more bets placed, more time and money spent gambling, and higher losses at the end of gambling sessions.

## Methods

### Data, sample, and procedure

Player account-based gambling data were obtained from *Norsk Tipping*, Norway's national gambling company and were retrieved from *Multix*, a video lottery terminal (VLT) operated by *Norsk Tipping*. *Multix* offers various games on its digital networked terminals. As an account-based system, *Multix* gamblers use personal player accounts providing the opportunity for anonymous behavioral tracking. Registered play is mandatory. The current dataset contained aggregated information about individuals' gambling behavior in venues (convenience stores, hotels, pubs etc.) with different numbers of gambling machines in 2015. The full dataset comprised 153,379 observations of 93,034 gamblers (age: 18–99 years, mean = 44.1, *SD* = 16.4 years) drawn by *Norsk Tipping*. Males comprised 74% of the sample. In 2015, there were 3,228 *Multix* terminals in 973 venues throughout Norway (*Norsk Tipping*, personal communication, 10.2.2017).

### Gambling measures

The operationalization of the measures analyzed in the present study is presented in Table 1.

**Table 1:** Operationalization of gambling measures.

**Measure**	**Operationalization**
Session	A period of continuous play from inserting the gambling card to retrieval
Time spent	Amount of time spent per session in seconds
Money spent	Amount of money spent per session in NOK
Bets placed	Number of bets placed per session
Net outcome	The difference between money spent and wins per session in NOK

### Statistical analysis

In the absence of an empirical basis or recommendation for grouping the number of gambling terminals present in venues, the following pragmatic categorization was used: venues with (a) only one terminal present, (b) two to five (2–5) terminals, (c) six to ten (6–10) terminals, and (d) eleven to sixteen (11–16) terminals. Compared to other gambling forms, EGM gambling is a form of gambling with a high event frequency where many bets can be placed within a short period of time (Harris and Griffiths, 2017). In-session gambling behavior is a common measure of gambling activity during EGM gambling sessions (e.g., duration, number of bets placed, net outcome including negative wins, and financial expenditure) (Leino et al., 2016, 2017). In the present study, average in-session gambling behavior per session (time spent, money spent, bets placed, and net outcome) was calculated by dividing an individual's overall gambling behavior in a venue category by his/her number of game sessions in that venue category (e.g., mean time spent per session in category = total time spent in category divided by the number of game sessions in category).

Due to the data's hierarchical and unbalanced structure, a linear mixed model (LMM) was specified. A LMM is a suitable analytical method for modeling heterogeneity among observations when the observations are not independent but clustered around a contextual factor and/or when there are uneven observations within different clusters (Hox, 2010; West et al., 2014). As such, a LMM was specified where within-session gambling behavior in venues with different numbers of EGMs (level 1) was nested within the individual (level 2).

Robust standard errors were used in the analysis to further account for any other unobserved heterogeneity not specified by the LMM. Due to non-normality and high variability, all variables (apart from financial losses) were log-transformed to improve their statistical properties. Net outcome was not transformed due to the presence of negative values from losses. To enhance the interpretability of the log-transformed variables, the values of the independent variables were exponentiated into percentage change relative to the reference categories (UCLA Statistical Consulting Group, 2017). The data were analyzed using Stata version 13 (StataCorp).

## Results

Table 2 presents overall individual gambling participation by categories of terminals. It shows that individuals gambled most frequently in venues with 2–5 terminals (54.5%) and least in venues with 11–16 terminals (1.6%). About half of the sample (52.5%) gambled in only one venue category, with majority of these (81.5%) preferring venues with 2–5 terminals present. Approximately one-third of the sample (31.0%) gambled in two venue categories with most switching between venues with 2–5 (49.1%) and 6–10 (36.6%) terminals present. Approximately one in six of the sample gambled in three different venue categories (15.8%) with almost all (97.8%) gambling in venues with one (31.4%), 2–5 (33.3%), and 6–10 (33.1%) terminals present. Only 0.8% of the sample had gambled in all the four venue categories.

**Table 2:** Individual gambling participation by categories of terminals.

**Terminals**	**1 category**^†^		**2 categories**^†^		**3 categories**^†^		**4 categories**^†^		***n***	**Row %**
	***n***	**%**	***n***	**%**	***n***	**%**	***n***	**%**		
1	1,748	3.6	7,678	13.3	13,813	31.4	723	25.0	23,962	15.6
2–5	39,792	81.5	28,329	49.1	14,670	33.3	723	25.0	83,514	54.5
6–10	7,023	14.4	21,107	36.6	14,548	33.1	723	25.0	43,401	28.3
11–16	244	0.5	550	1.0	985	2.2	723	25.0	2,502	1.6
*N* and column %	48,807	52.5	28,832	31.0	14,672	15.8	723	0.78	153,379	100

*N, Individual gamblers = 93,034; n, unique observations = 153,379*.

† *Gambled in…; Column %, proportion of unique individuals in a column; Row %, proportion of unique observations in a row*.

Table 3 shows descriptive statistics of overall gambling behavior in venues with different numbers of terminals over a year. There were curvilinear relationships (increases from one terminal only to 2–5 terminals and subsequent decreases from 6–10 terminals to 11–16 terminals) between gambling measures (days gambled, number of sessions, bets placed, time and money spent, and net outcome) and the number of terminals in a venue.

**Table 3:** Average overall gambling behavior in venues with different numbers of gambling terminals.

**Measure**	**1 terminal**				**2–5 terminals**				**6–10 terminals**				**11–16 terminals**			
	**Mean**	***SD***	**Md**	**MAD**	**Mean**	***SD***	**Md**	**MAD**	**Mean**	***SD***	**Md**	**MAD**	**Mean**	***SD***	**Md**	**MAD**
Days gambled^§^	7.9	16.4	2.0	1.0	30.6	42.3	10.0	9.0	22.2	34.2	7.0	6.0	17.1	30.6	4.0	3.0
Sessions^§^	23.7	73.3	5.0	4.0	138.3	306.5	27.0	26.0	105.6	247.1	20.0	18.0	96.4	232.2	14.0	12.0
Bets placed^§^	1,338.8	4,001.6	249.0	221.0	7,820.6	14,590.5	1,383.0	1,358.0	6,166.1	12,693.1	1,125.0	1,080.0	5,130.6	10,593.5	756.0	703.5
Time spent (s)^§^	7,803.1	22,868.7	1,493.0	1,310.0	45,410.5	83,594.1	8,523.5	8,310.5	35,750.6	72,127.7	6,711.0	6,404.0	29,198.2	60,273.0	4,391.5	4,081.0
Money spent (NOK)^§^	10,161.7	26,697.6	1,929.0	1,751.0	56,340.1	89,886.2	10,887.0	10,744.3	43,965.8	75,626.7	8,976.0	8,682.0	35,054.5	66,391.1	5,813.0	5,446.5
Net outcome (NOK)	−787.5	2,389.8	−275.0	325.0	−4,425.5	7,186.7	−989.8	1,126.1	−3,491.5	6,238.8	−833.0	990.1	−2,738.3	5,478.1	−600.0	748.1

§ *Log. Md, Median; MAD, Median absolute deviation; N, individual gamblers = 93,034; n, unique observations = 153,379*.

Table 4 shows descriptive statistics of average gambling behavior per gambling session. There was a discrepancy between results from mean and median values. The mean values indicated that individuals gambled least (bets placed, and time and money spent) in venues with 2–5 terminals. However, median values indicated that gambling (bets placed, and time and money spent) increased with a greater number of terminals in a venue. Nonetheless, both mean and median values show that venues with 2–5 terminals were associated with the lowest losses.

**Table 4:** Average gambling behavior per session in venues with different numbers of gambling terminals.

**Measure**	**1 terminal**				**2–5 terminals**				**6–10 terminals**				**11–16 terminals**			
	**Mean**	***SD***	**Md**	**MAD**	**Mean**	***SD***	**Md**	**MAD**	**Mean**	***SD***	**Md**	**MAD**	**Mean**	***SD***	**Md**	**MAD**
Bets placed^§^	60.0	57.4	44.3	23.4	56.8	46.6	46.2	23.4	63.6	50.8	51.9	23.6	66.2	54.3	52.0	23.1
Time spent (s)^§^	355.3	336.1	262.1	136.2	350.4	273.4	285.9	130.4	381.2	299.1	310.7	135.7	382.4	319.4	296.1	131.9
Money spent (NOK)^§^	518.5	604.6	337.8	217.8	479.7	477.6	348.3	219.3	538.3	530.3	395.0	230.2	550.7	549.2	389.3	220.5
Net outcome (NOK)	−62.9	217.6	−50.6	52.7	−54.1	121.1	−40.7	32.3	−62.2	157.6	−47.4	40.9	−66.9	197.4	−50.0	47.0

§ *Log. Md, Median; MAD, Median absolute deviation; N, individual gamblers = 93,034; n, unique observations = 153,379*.

Table 5 shows results from the mixed effects regression model. The model for number of bets placed was significant: Wald $\chi_{\left(3\right)}^{2}$ = 623.39, *p* < 0.001. Compared to venues with one terminal, individuals placed more bets in venues with 2–5 (Δ = 0.09, CI95% [0.08, 0.10]), 6–10 (Δ = 0.12, CI95% [0.11, 0.13]), and 11–16 (Δ = 0.10, CI95% [0.06, 0.13]) terminals. This shows that *if* individuals placed, on average, 100 bets in venues with only one terminal, they placed 9 (9%), 12 (12%), and 10 (10%) more bets in venues with 2–5, 6–10, and 11–16 terminals respectively. Additionally, more bets were placed in venues with 6–10 terminals compared to venues with 2–5 terminals (Δ = 0.03, CI95% [0.02, 0.04]). Hence, 3 (3%) more bets were placed in venues with 6–10 terminals compared to venues with 2–5 terminals in relation to the above example. No differences in bets placed were observed between venues with 2–5 and 11–16 terminals (Δ = 0.00, CI95% [−0.03, 0.03]), or between venues with 6–10 and 11–16 terminals (Δ = −0.03, CI95% [−0.06, 0.00]).

**Table 5:** Results from a mixed effects model predicting gambling behavior in venues with different numbers of gambling terminals (fixed effects).

**Measure**	**1 terminal**		**2–5 terminals**		**6–10 terminals**		**11–16 terminals**		**Comparison**	
	**Mean**	**95% CI**	**Mean**	**95% CI**	**Mean**	**95% CI**	**Mean**	**95% CI**	**Wald χ2**^†^	***p* <**
Bets placed^§^	3.60	[3.59, 3.61]	3.69^a^	[3.68, 3.70]	3.72^b^	[3.71, 3.73]	3.69^a b^	[3.67, 3.72]	623.39	0.001
Time spent (s)^§^	5.44	[5.43, 5.45]	5.57^a^	[5.56, 5.58]	5.59	[5.59, 5.60]	5.55^a^	[5.52, 5.58]	833.86	0.001
Money spent (NOK)^§^	5.55	[5.54, 5.57]	5.67^a^	[5.66, 5.68]	5.69	[5.68, 5.70]	5.65^a^	[5.61, 5.68]	544.09	0.001
Net outcome (NOK)	−63.24^a^	[−66.75, −59.73]	−54.09	[−55.14, −53.05]	−62.19^a^	[−64.08, −60.30]	−66.85^a^	[−76.70, −57.01]	124.71	0.001

§ *Log*.

† *df = 3. N, individual gamblers = 93,034; n, unique observations = 153,379*.

*Figures sharing a letter are NOT different (p > 0.05)*.

*Results are Bonferroni corrected*.

Moreover, there was a significant model for time spent: Wald $\chi_{\left(3\right)}^{2}$ = 833.86, *p* < 0.001. Compared to venues with one terminal, individuals spent more time in venues with 2–5 (Δ = 0.13, CI95% [0.11, 0.14]), 6–10 (Δ = 0.15, CI95% [0.14, 0.17]), and 11–16 (Δ = 0.11, CI95% [0.07, 0.14]) terminals. This indicates that *if* individuals gambled on average 600 s in venues with only one terminal, they spent 13% (78 s), 15% (90 s), and 11% (66 s) more time in venues with 2–5, 6–10, and 11–16 terminals respectively. More time was spent in venues with 6–10 terminals compared to venues with 2–5 (Δ = 0.02, CI95% [0.02, 0.03]), and 11–16 (Δ = 0.05, CI95% [0.01, 0.08]) terminals. That is, compared to venues with 6–10 terminals, 2% (12 s) more time was spent in venues with 2–5 terminals whereas 5% (30 s) more time was spent in venues with 11–16 terminals in line with the example above. No difference in time spent was observed between venues with 2–5 and 11–16 terminals (Δ = − 0.02, CI95% [−0.05, 0.01]).

The model for money spent was also significant: Wald $\chi_{\left(3\right)}^{2}$ = 544.09, *p* < 0.001. Compared to venues with one terminal, individuals spent significantly more money in venues with 2–5 (Δ = 0.11, CI95% [0.10, 0.13]), 6–10 (Δ = 0.14, CI95% [0.12, 0.16]), and 11–16 (Δ = 0.10, CI95% [0.06, 0.14]) terminals. Hence, *if* individuals spent on average NOK 100 (≈ € 10.45) in venues with only one terminal, they spent NOK 11 (11%), NOK 14 (14%), and NOK 10 (10%) more in venues with 2–5, 6–10, and 11–16 terminals respectively. Venues with 6–10 terminals were associated with most money spent, significantly different from venues with 2–5 (Δ = 0.03, CI95% [0.02, 0.03]), and 11–16 (Δ = 0.04, CI95% [0.01, 0.08]) terminals. Thus, compared to venues with 6–10 terminals, venues with 2–5 terminals were associated with NOK 3 (3%) less spent whereas venues with 11–16 terminals were associated with NOK 4 (4%) less spent consistent with the above example. No difference in money spent was observed between venues with 2–5 and 11–16 terminals (Δ = −0.02, CI95% [−0.05, 0.02]).

Further, the model for net outcome was significant: Wald $\chi_{\left(3\right)}^{2}$ = 124.71, *p* < 0.001. Compared to venues with 2–5 terminals, gamblers had higher losses in venues with one (Δ = −9.15, CI95% [5.32, 12.98]), 6–10 (Δ = −8.10, CI95% [−10.33, −5.87]), and 11–16 (Δ = −12.76, CI95% [−23.21, −2.31]) terminals. Therefore, compared to venues with 2–5 terminals, on average, individuals lost NOK 9.15 (≈ € 0.96), NOK 8.10 (≈ € 0.85), and NOK 12.76 (≈ € 1.33) more (money) in venues with only one, 6–10, and 11–16 terminals respectively. However, compared to venues with one terminal, no significant differences in net outcome were found in venues with 6–10 (Δ = 1.05, CI95% [−3.11, 5.22]), and 11–16 (Δ = −3.61, CI95% [−14.66, 7.44]) terminals. No differences in net outcome were found between venues with 6–10 and 11–16 (Δ = −4.66, CI95% [−15.24, 5.92]) terminals.

## Discussion

Using the number of gaming terminals per venue as an environmental factor or proxy for congregations of gamblers, player account-based gambling data were analyzed to compare individual gambling behavior across venues with differing numbers of gambling terminals.

A plausible explanation for our finding that individuals most frequented venues with 2–5 terminals (54.5%) and least frequented venues with 11–16 terminals (1.6%) is that venues with larger numbers of terminals are more likely to be perceived by individuals as “dedicated gambling venues” and are therefore more attractive to “dedicated gamblers” (Young et al., 2012; Sévigny et al., 2016). In contrast, gambling is more likely to be peripheral to the activities of venues with smaller numbers of terminals and thus more likely to be socially accessible and attractive to the general population of both “dedicated” and occasional gamblers (Doran and Young, 2010; Hing and Haw, 2010).

Additionally, our finding that approximately half of the sample (52.5%) gambled in only one venue category is consistent with evidence that a large proportion of gamblers (especially problem gamblers) have favorite venues and EGMs with some leaving the venue if unable to play on their favorite EGM, while others wait and sometimes try other EGMs until they can play on their favorite (AIPC, 2006; Hing and Haw, 2010). It is also tenable that the 15.8 and 0.8% who had gambled in three and all four venue categories respectively reflect individuals who gamble regularly or perceive themselves as “gamblers” and therefore take the opportunity to gamble irrespective of the primary purpose of the venue or the number of terminals available. It should be noted that gambling in multiple venues has been associated with gambling problems (Franco et al., 2011; Sévigny et al., 2016).

Consistent with expectations, findings demonstrated variability in individual gambling behavior in relation to the number of available terminals. Overall, compared to venues with one terminal, venues with two or more terminals were associated with gamblers placing higher bets, and spending more time and money per session. Generally, these findings are in line with evidence from survey studies showing that, compared to smaller venue gamblers, larger venue gamblers have higher monetary expenditure per visit (Haw and Hing, 2011), and gambling time per session (Young et al., 2012; Sévigny et al., 2016).

Findings are also consistent with experimental studies supporting the gambling environment's (e.g., co-action and audiovisual feedback) reinforcement of gambling behavior (Rockloff and Dyer, 2007; Cole et al., 2011; Brevers et al., 2015) and with observational studies indicating that gamblers play longer when with friends (Griffiths and Parke, 2003), although contrary evidence has been reported on average trials gambled and gambling speed (Molde et al., 2017). As suggested previously, methodical approaches (e.g., experiments vs. surveys) and analytical differences (e.g., categorization of venue size/terminal numbers) in previous studies may account for the inconsistent findings.

It is not clear why venues with one terminal were associated with higher losses (even if small: NOK 9.15, ≈ € 0.96) compared to venues with 2–5 terminals, and why no differences in net outcome were found between venues with one terminal and those with 6–10 and 11–16 terminals. However, it is tenable that in contrast to the other study variables (gambling venue, days gambled, number of sessions, bets placed, and time and money spent), net outcomes such as wins and losses are beyond the control of gamblers. For instance, gamblers may have played different games as a function of the number of terminals. As such, the unique structural characteristics of games such as bet and reward characteristics (Parke and Griffiths, 2007; Leino et al., 2015, 2016) might have influenced their net outcome.

Taken as a whole, the present findings provide novel evidence from the natural gambling environment that gambling behavior is reinforced in venues with multiple terminals (compared to venues with only one terminal). It is reasonable that the presence of other gamblers in large venues or venues with multiple terminals more commonly promotes and broadcasts individual wins to others thereby reinforcing and normalizing gambling intensity (Rockloff et al., 2016) or that the presence of friends helps prolong gambling sessions (Griffiths and Parke, 2003). An alternative explanation for the present findings is deducible from the integrated behavioral model (Fishbein, 2000, 2008). For instance, it is possible that the presence of multiple terminals (and possibly other active gamblers) serves as a distal variable that promotes positive beliefs and intentions about gambling by influencing gambling-related attitudes, subjective norms, and self-efficacy. Given the relative simplicity of slot machine gambling and the environmental cues or inducements provided by the presence of multiple terminals (and possibly other active gamblers), gambling behavior is then facilitated. Furthermore, the present findings could be explained by pre-existing individual differences in gamblers who prefer playing in venues with one vs. multiple terminals.

### Strengths, limitations, and directions for future research

As far as we are aware, the present study is the first to compare individual gambling behavior across venues with different numbers of gambling terminals, in the natural gambling environment. The large number of gamblers and observations are assets of the present study. Also, the study's reliance on player account-based gambling data overcomes ecological challenges, observer-expectancy effects, and subject-expectancy effects associated with experimental or laboratory studies (Griffiths, 2014). It also overcomes reporting accuracy and selective venues monitored in observational studies.

Nonetheless, it must be noted that Norway's legal regulations such as limits on venue size (e.g., no casinos are permitted), and structural limits such as maximum play time as well as win and loss limits restrict real-world gambling and could have influenced the present findings. Also, between-category differences in losses were relatively small (e.g., one terminal vs. 2–5 terminals: NOK 9.15, ≈ € 0.96) and may thus not reflect real-world differences. Another limitation of the present study is the assumption that the number of gambling terminals available in a gambling venue is a good proxy for numbers of gamblers in the venues. In addition to the number of terminals in a venue, factors such as the type of venue (e.g., cafeteria, grocery store, hotel, and pub), servicescape features such as the amount of audio-visual stimuli and availability of refreshment (Griffiths, 2009), as well as player intent, time of day and season, and the degree of socialization during gambling sessions may also have influenced individual variation of gambling behavior across venues with different numbers of gambling terminals.

Moreover, as indicated previously, our categorization of the number of gambling terminals present in venues was pragmatic due to the absence of anything empirical in the current literature. We placed venues with one terminal in a separate category as this stands in contrast to other venues in terms of the availability of multiple terminals and potential influence of co-action. It should also be noted that specific venue characteristics might have appealed to specific subtypes of gamblers (Hing and Haw, 2010). Hence, both unique individual and venue characteristics may have acted as confounders in the present study. The aforementioned individual and environmental characteristics variables were not available for the present analysis. Although difficult to “behaviorally-track” (Griffiths, 2014), future studies using player account-based gambling data are encouraged to examine the such factors and others described elsewhere (Hing and Haw, 2010; Haw and Hing, 2011; Sévigny et al., 2016) where accessible.

## Conclusion

The present study extends previous experimental laboratory, survey, and observational investigations that have examined individual differences in gambling behavior in gambling venues with different numbers of gambling terminals. The study's use of player account-based gambling data overcomes methodical and ecological limitations associated with previous studies. Based on data from the natural gambling environment, the present study demonstrates that gambling behavior is strengthened in venues with multiple gambling terminals.

## Ethics statement

A contract between the University of Bergen (UiB) and *Norsk Tipping* was signed in June 2011, which permits UiB to use *Norsk Tipping's* data in anonymous form for research purposes. Gamblers provided consent in their personal contracts with *Norsk Tipping*. In order to ensure anonymity, *Norsk Tipping* created new identity codes for each participant and deleted the keys to the new identity codes prior to data export and delivery. Apart from supplying the anonymous data, *Norsk Tipping* played no role in the scientific and dissemination process and made no financial contribution to the project. As we conducted analysis of anonymized behavioral tracking data, obtaining ethical approval was not necessary (Bruin-Huisman et al., 2017). The study was conducted in line with the Declaration of Helsinki.

## Author contributions

SP designed and obtained funding for the study. TL conducted the statistical analyses. DS drafted the initial manuscript. All authors contributed to the writing process and approved the final manuscript.

### Conflict of interest statement

The author MG has received funding for a number of research projects in the area of gambling education for young people, social responsibility in gambling and gambling treatment from Gamble Aware (formerly the Responsibility in Gambling Trust), a charitable body which funds its research program based on donations from the gambling industry. The author MG also undertakes consultancy for various gaming companies in the area of social responsibility in gambling. The other authors declare that the research was conducted in the absence of any commercial or financial relationships that could be construed as a potential conflict of interest. The reviewer SH and handling editor declared their shared affiliation.
